# Crystal structure and Hirshfeld surface analysis of 4-(4-methyl­benz­yl)-6-phenyl­pyridazin-3(2*H*)-one

**DOI:** 10.1107/S2056989019011551

**Published:** 2019-08-23

**Authors:** Said Daoui, Emine Berrin Cinar, Fouad El Kalai, Rafik Saddik, Khalid Karrouchi, Noureddine Benchat, Cemile Baydere, Necmi Dege

**Affiliations:** a Laboratory of Applied Chemistry and Environment (LCAE), Department of Chemistry, Faculty of Sciences, University Mohamed Premier, Oujda 60000, Morocco; b Ondokuz Mayıs University, Faculty of Arts and Sciences, Department of Physics, Kurupelit, Samsun, Turkey; c Laboratory of Organic Synthesis, Extraction and Development, Faculty of Sciences, Hassan II University, Casablanca, Morocco; d Laboratory of Plant Chemistry, Organic and Bioorganic Synthesis, URAC23, Faculty of Science, BP 1014, GEOPAC Research Center, Mohammed V University, Rabat, Morocco

**Keywords:** crystal structure, Hirshfeld surface analysis, pyridazine, pyridazine derivative, pyridazinone

## Abstract

In this paper, we describe the synthesis of a new di­hydro-2*H*-pyridazin-3-one derivative. The mol­ecule, C_18_H_16_N_2_O, is not planar; the benzene and pyridazine rings are twisted with respect to each other, making a dihedral angle of 11.47 (2)°, and the toluene ring is nearly perpendicular to the pyridazine ring, with a dihedral angle of 89.624 (1)°. The mol­ecular conformation is stabilized by weak intra­molecular C—H⋯N contacts. In the crystal, pairs of N—H⋯O hydrogen bonds link the mol­ecules into inversion dimers with an *R*_2_^2^(8) ring motif. The inter­molecular inter­actions were investigated using Hirshfeld surface analysis and two-dimensional (2D) fingerprint plots, revealing that the most important contributions for the crystal packing are from H⋯H (56.6%), H⋯C/C⋯H (22.6%), O⋯H/H⋯O (10.0%) and N⋯C/C⋯N (3.5%) inter­actions.

## Abstract 2

In the title compound, inter­molecular N—H⋯O hydrogen bonds link the mol­ecules into a three-dimensional supra­molecular network.

## Chemical context   

Pyridazines are an important family of six-membered aromatic heterocycles containing two N atoms. Pyridazinone is an important pharmacophore possessing a wide range of biological applications (Asif, 2014 ▸; Akhtar *et al.*, 2016 ▸). The chemistry of pyridazinones has been an inter­esting field of study for decades and this nitro­gen heterocycle has become a scaffold of choice for the development of potential drug candidates (Dubey & Bhosle, 2015 ▸; Thakur *et al.*, 2010 ▸). A review of the literature has revealed that substituted pyridazinones have received a lot of attention in recent years because of their significant potential as anti­microbial (Sönmez *et al.*, 2006 ▸), anti­depressant (Boukharsa *et al.*, 2016 ▸), anti-inflammatory (Barberot *et al.*, 2018 ▸), anti­hypertensive (Siddiqui *et al.*, 2011 ▸), analgesic (Gökçe *et al.*, 2009 ▸), anti-HIV (Livermore *et al.*, 1993 ▸), anti­convulsant (Partap *et al.*, 2018 ▸; Sharma *et al.*, 2014 ▸), cardiotonic (Wang *et al.*, 2008 ▸), anti­histaminic (Tao *et al.*, 2012 ▸), glucan synthase inhibitors (Zhou *et al.*, 2011 ▸), phospho­diesterase (PDE) inhibitors (Ochiai *et al.*, 2012 ▸) and herbicidal agents (Asif, 2013 ▸). In continuation of our work in this field (El Kali *et al.*, 2019 ▸; Chkirate *et al.*, 2019*a* ▸,*b* ▸; Karrouchi *et al.*, 2015 ▸, 2016*a* ▸,*b* ▸), we report the synthesis and the crystal and mol­ecular structures of the title compound, as well as an analysis of its Hirshfeld surface.

## Structural commentary   

**Figure 1:**
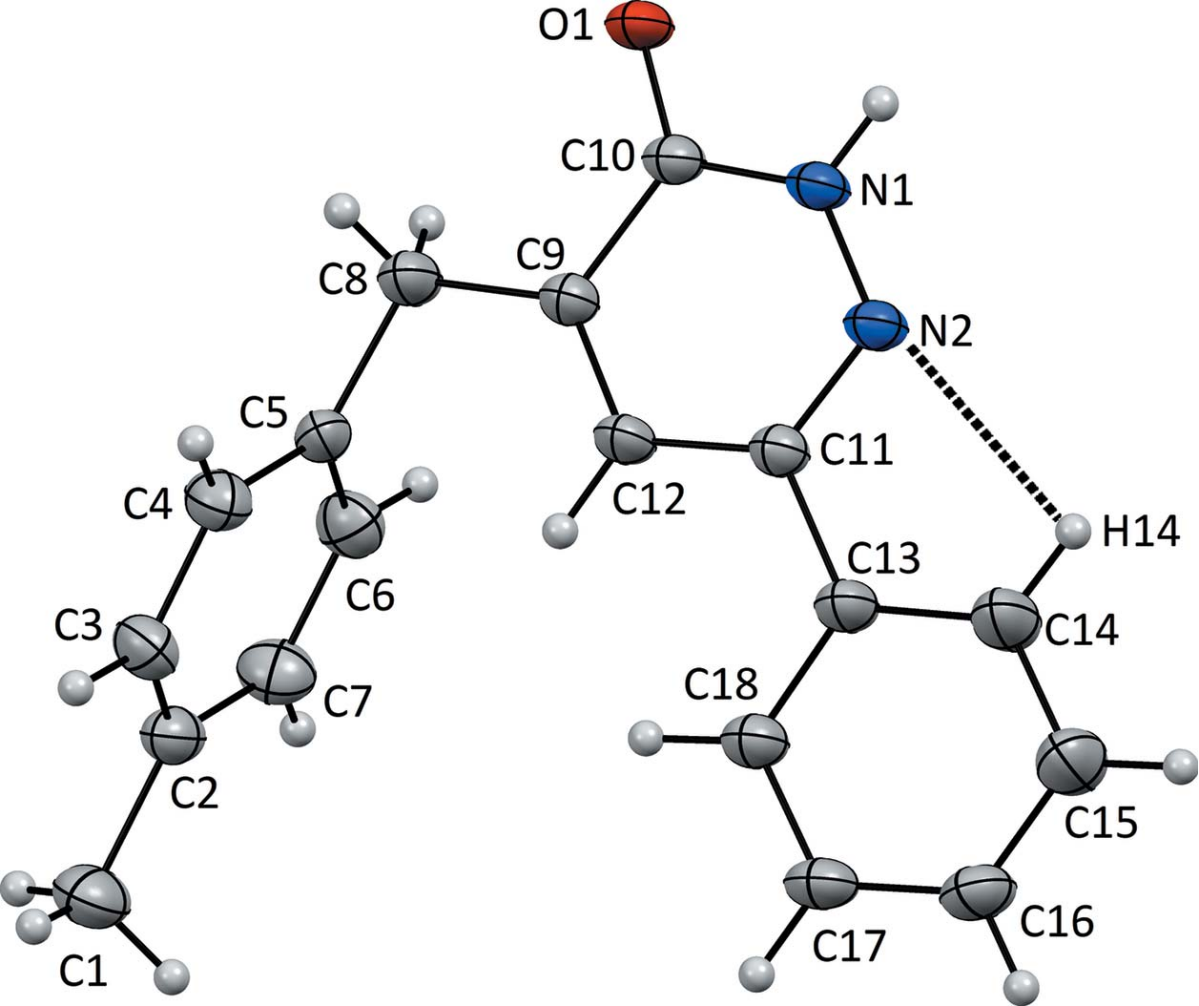
The mol­ecular structure of the title compound, with the atom labelling. Displacement elipsoids are drawn at the 20% probability level.

In the title mol­ecule (Fig. 1 ▸), the C10=O1 bond length is 1.241 (3) Å while the N1—N2 and C11=N2 bond lengths are 1.347 (3) and 1.311 (4) Å, respectively (Table 1 ▸). The C9—C8—C5 bond angle is 113.7 (2)°, while the C4—C5—C8—C9, C6—C5—C8—C9 and C10—C9—C8—C5 torsion angles are 90.0 (3), −87.1 (3) and 169.1 (3)°, respectively. The mol­ecule is not planar as the benzene and pyridazine rings are twisted with respect to each other, making a dihedral angle of 11.469 (2)°. The toluene ring is nearly perpendicular to the pyridazine ring, with a dihedral angle of 89.624 (1)°.
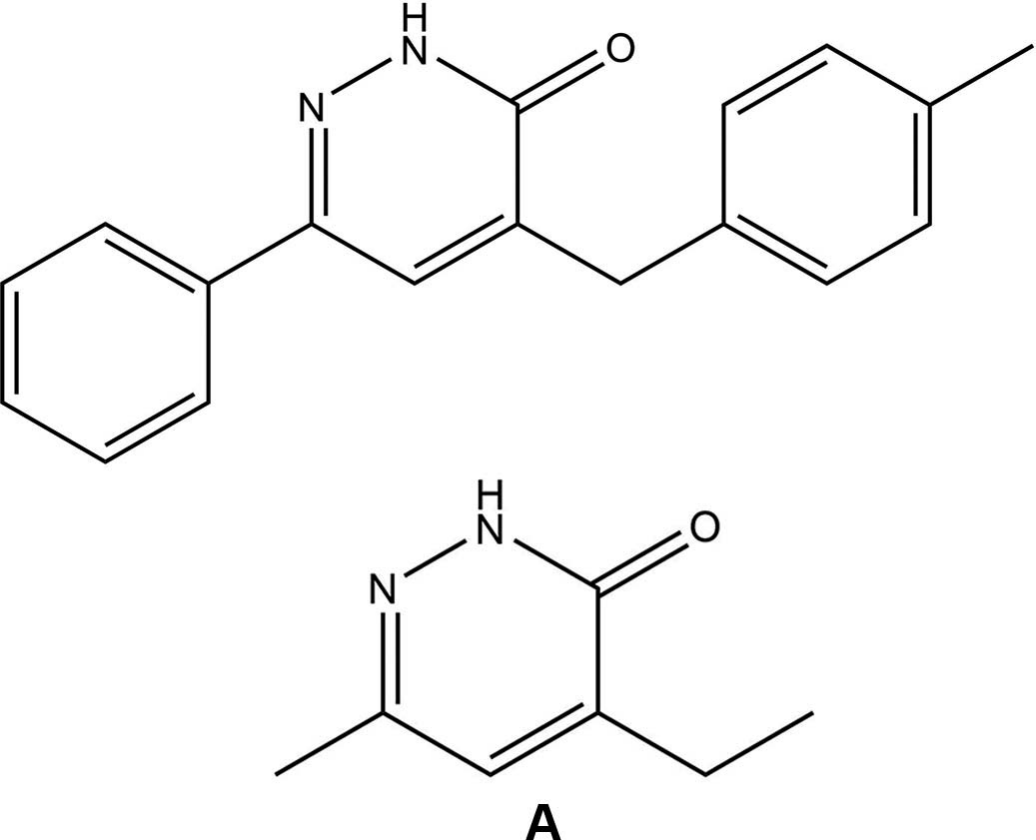


## Supra­molecular features   

**Figure 2:**
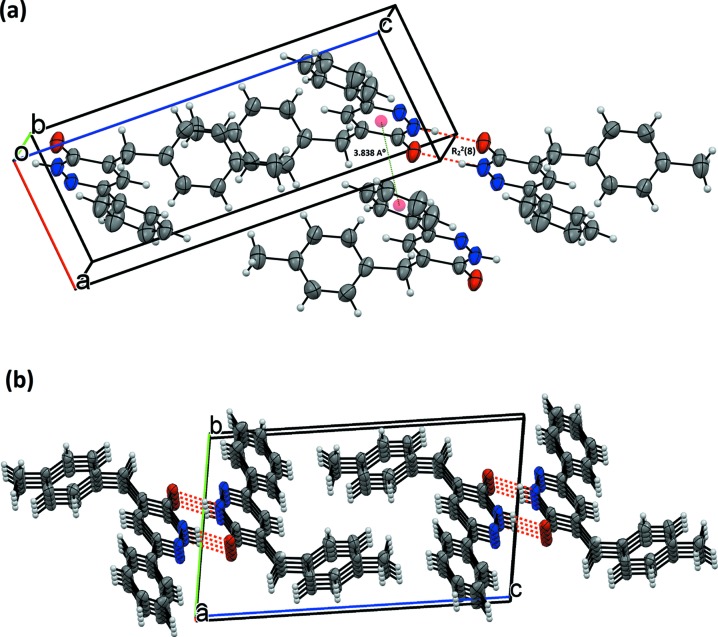
(*a*) A view along the *c*-axis direction of the title structure. Red dashed lines denote N—H⋯O hydrogen bonds. (*b*) A view along the *a*-axis direction of the title compound (Xu *et al.*, 2005 ▸).

The mol­ecules are connected two-by-two through N1—H1⋯O1 hydrogen bonds (Table 2 ▸), with a 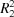
(8) graph-set motif (Bernstein *et al.*, 1995 ▸), and form inversion dimers (Fig. 2 ▸ *a*). Weak C—H⋯O hydrogen bonds and weak off-set π-stacking stabilize the packing. In the crystal, hydrogen bonds link the chains into a two-dimensional (2D) network parallel to (011) (Fig. 2 ▸ *b* and Table 2 ▸). The stacking occurs between the pyridazine rings of inversion-related mol­ecules [*Cg*1⋯*Cg*3 (at *x* − 1, *y*, *z*)], with a centroid-to-centroid distance of 3.8333 (18) Å and a slippage of 1.460 Å (*Cg*1 is the centroid of the C9–C11/N1/N2 ring and *Cg*3 is the centroid of the C13–C18 ring) (Fig. 2 ▸ *a*).

## Database survey   

A search of the Cambridge Structural Database (CSD, Version 5.40, update of November 2018; Groom *et al.*, 2016 ▸) using 4-ethyl-6-methyl­pyridazin-3(2*H*)-one (see **A** in Scheme[Chem scheme1]) as the main skeleton revealed the presence of two structures containing the pyridazine moiety with different substituents similar to the title compound in this study. The structures are 4-benzyl-6-*p*-tolyl­pyridazin-3(2*H*)-one (CSD refcode YOT­VIN; Oubair *et al.*, 2009 ▸) and 4-aryl-2,5-dioxo-8-phenyl­pyrido[2,3-*d*]pyridazines (BARQUG; Pita *et al.*, 2000 ▸). In YOTVIN, the mol­ecules are connected two-by-two through N—H⋯O hydrogen bonds, with an 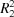
(8) graph-set motif, building a pseudo-dimer arranged around the inversion centre. Weak C—H⋯O hydrogen bonds and weak off-set π–π stacking stabilize the packing. In BARQUG, the dihedral angle between the least-squares planes of the substituted phenyl and pyridone rings is 79.78 (2)° and between the pyridazinone ring and the unsubstitued phenyl ring is 57.37 (2)°.

## Hirshfeld surface (HS) analysis   

**Figure 3:**
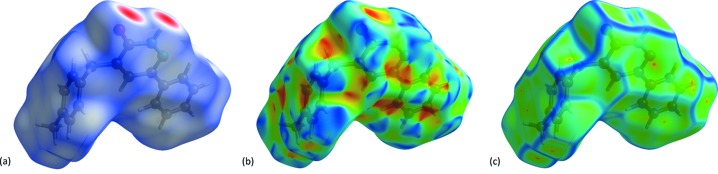
(*a*) *d*_norm_ mapped on the Hirshfeld surface for visualizing the inter­molecular inter­actions; (*b*) shape-index map; (*c*) curvedness map of the title compound.

**Figure 5:**
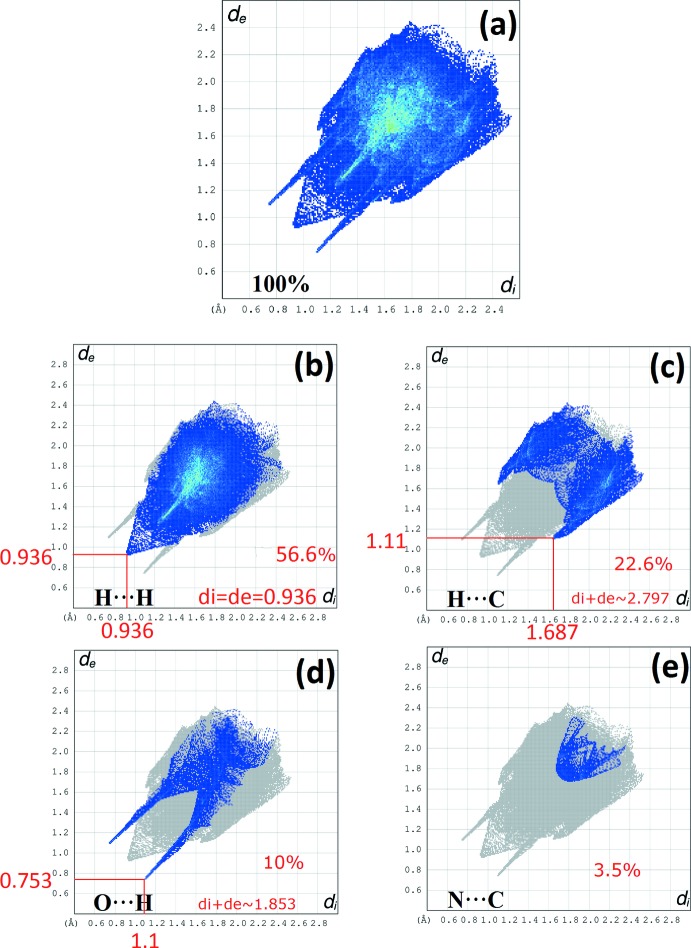
(*a*) The overall 2D fingerprint plot and (*b*) H⋯H, (*c*) C⋯H, (*d*) O⋯H and (*e*) N⋯C inter­actions are shown.

**Figure 6:**
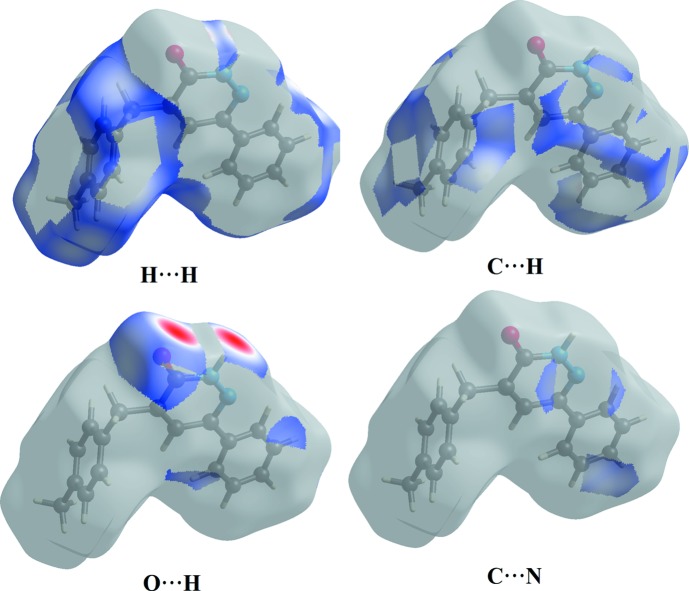
Hirshfeld surface representation with the function *d*_norm_ plotted on the surface for H⋯H, C⋯H, O⋯H and N⋯C inter­actions.

The Hirshfeld surface analysis (Spackman & Jayatilaka, 2009 ▸) and the associated 2D fingerprint plots (McKinnon *et al.*, 2007 ▸) were performed with *CrystalExplorer17* (Turner *et al.*, 2017 ▸). The Hirshfeld surface was calculated using a standard (high) surface resolution with the three-dimensional (3D) *d*_norm_ surface plotted over a fixed colour scale of −0.6048 (red) to 1.4188 a.u. (blue). The 3D *d*_norm_ surface of the title complex is illustrated in Figs. 3 ▸(*a*) and 4 ▸. The pale-red spots symbolize short contacts and negative *d*_norm_ values on the surface correspond to the N—H⋯O inter­actions (Table 2 ▸). The overall 2D fingerprint plot and the 2D fingerprint plots for the H⋯H, H⋯C/C⋯H, H⋯O/O⋯H and N⋯C/C⋯N contacts are shown in Fig. 5 ▸ (McKinnon *et al.*, 2007 ▸), respectively, associated with their relative contributions to the Hirshfeld surface. The largest inter­action is H⋯H, contributing 56.6% to the overall crystal packing. In the fingerprint plot representing H⋯H contacts, the 56.6% contribution to the overall crystal packing, is reflected by widely scattered points of high density due to the large hydrogen content of the mol­ecule. The single spike in the centre at *d*_e_ = *d*_i_ = 0.936 Å in Fig. 5 ▸(*b*) is due to short inter­atomic H⋯H contacts. In the absence of C—H⋯π inter­actions in the crystal, the pair of characteristic wings in the fingerprint plot representing H⋯C/C⋯H contacts (22.6% contribution to the HS) have a symmetrical distribution of points (Fig. 5 ▸ *c*), with the tips at *d*_e_ + *d*_i_ = 2.797 Å. The O⋯H (Fig. 5 ▸ *d*) contacts contribute 10% to the HS and have a symmetrical distribution of points, with the tips at *d*_e_ + *d*_i_ = 1.853 Å. The contribution of the other contact to the Hirshfeld surface is N⋯C/C⋯N (3.5%). The Hirshfeld surface representations with the function *d*_norm_ plotted on the surface are shown for the H⋯H, H⋯C/C⋯H, H⋯O/O⋯H, C⋯C and H⋯N/N⋯H inter­actions in Figs. 6 ▸. The Hirshfeld surface analysis confirms the importance of H-atom contacts in establishing the packing. The large number of H⋯H, H⋯C/C⋯H, H⋯O/O⋯H, C⋯C and H⋯N/N⋯H inter­actions suggest that van der Waals inter­actions and hydrogen bonding play the major roles in the crystal packing (Hathwar *et al.*, 2015 ▸).

A shape-index map of the title compound was generated in the range −1 to 1 Å (Fig. 3 ▸ *b*). The convex blue regions on the shape-index symbolize hydrogen-donor groups and the concave red regions symbolize hydrogen-acceptor groups. The π–π inter­actions on the shape-index map of the Hirshfeld surface are generally indicated by adjacent red and blue triangles.

A curvedness map of the title compound was generated in the range −4 to 0.4 Å (Fig. 3 ▸ *c*). This shows large regions of green indicating a relatively flat surface area (planar), while the blue regions indicate areas of curvature. The presence of π–π stacking inter­actions is also evident in the flat regions around the rings on the Hirshfeld surface plotted over curvedness (see the *Supra­molecular features* section above).

## Synthesis and crystallization   

To a solution (0.15 g, 1 mmol) of 6-phenyl-4,5-di­hydro­pyridazin-3(2*H*)-one and (0.12 g, 1 mmol) of 4-methyl­benz­aldehyde in ethanol (30 ml), sodium hydroxide (10%, 0.5 g, 3.5 mmol) was added. The solvent was evaporated under vacuum and the residue was purified through silica-gel column chromatography using hexa­ne/ethyl acetate (7:3 *v*/*v*). Slow evaporation at room temperature leads to single crystals.

## Refinement   

H atoms were fixed geometrically and treated as riding, with C—H = 0.97 Å and *U*_iso_(H) = 1.5*U*_eq_(C) for methyl, C—H = 0.96 Å and *U*_iso_(H) = 1.2*U*_eq_(C) for methyl­ene, C—H = 0.93 Å and *U*_iso_(H) = 1.2*U*_eq_(C) for aromatic and C—H = 0.98 Å and *U*_iso_(H) = 1.2*U*_eq_(C) for methine H atoms. Crystal data, data collection and structure refinement details are summarized in Table 3 ▸.

## Supplementary Material

Crystal structure: contains datablock(s) I. DOI: 10.1107/S2056989019011551/mw2146sup1.cif

Structure factors: contains datablock(s) I. DOI: 10.1107/S2056989019011551/mw2146Isup3.hkl

CCDC reference: 1947718

Additional supporting information:  crystallographic information; 3D view; checkCIF report

## Figures and Tables

**Figure 4:**
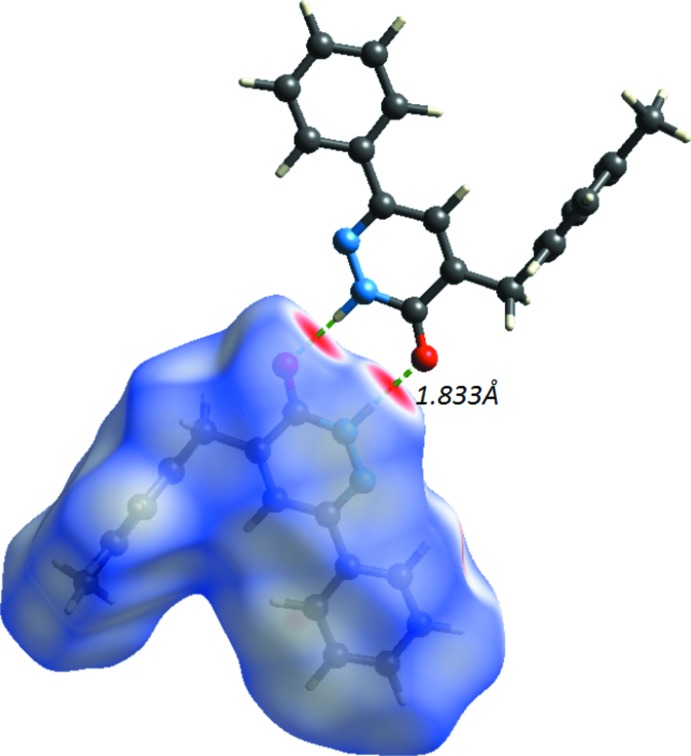
*d*_norm_ mapped on the Hirshfeld surface for visualizing the inter­molecular inter­actions and showing the dimer formed by inversion-related N—H⋯O hydrogen bonds.

**Table 1:** Selected geometric parameters (Å, °)

O1—C10	1.241 (3)	N1—C10	1.352 (4)
N1—N2	1.347 (3)	N2—C11	1.311 (4)
			
O1—C10—N1	120.9 (3)	C10—C9—C8	117.5 (2)
O1—C10—C9	123.9 (3)	C9—C8—C5	113.7 (2)
			
N1—N2—C11—C13	177.4 (3)	C4—C5—C8—C9	90.3 (4)
C10—C9—C8—C5	169.2 (3)	C6—C5—C8—C9	−86.8 (4)

**Table 2:** Hydrogen-bond geometry (Å, °)

*D*—H⋯*A*	*D*—H	H⋯*A*	*D*⋯*A*	*D*—H⋯*A*
N1—H1⋯O1^i^	0.86	1.98	2.836 (3)	175
C14—H14⋯N2	0.93	2.43	2.764 (3)	101

Symmetry code: (i) 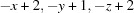.

**Table 3:** Experimental details

Crystal data	
Chemical formula	C_18_H_16_N_2_O
*M*_r_	276.33
Crystal system, space group	Triclinic, *P* 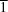
Temperature (K)	296
*a*, *b*, *c* (Å)	5.8479 (5), 8.5738 (7), 15.2439 (12)
α, β, γ (°)	80.693 (6), 83.147 (7), 78.164 (7)
*V* (Å^3^)	735.27 (11)
*Z*	2
Radiation type	Mo *K*α
μ (mm^−1^)	0.08
Crystal size (mm)	0.27 × 0.20 × 0.06

Data collection	
Diffractometer	Stoe IPDS 2
Absorption correction	Integration (*X-RED32*; Stoe & Cie, 2002 ▸)
*T*_min_, *T*_max_	0.966, 0.996
No. of measured, independent and observed [*I* > 2σ(*I*)] reflections	9453, 2887, 1471
*R*_int_	0.086
(sin θ/λ)_max_ (Å^−1^)	0.617

Refinement	
*R*[*F* ^2^ > 2σ(*F* ^2^)], *wR*(*F* ^2^), *S*	0.068, 0.208, 1.05
No. of reflections	2887
No. of parameters	191
No. of restraints	84
H-atom treatment	H-atom parameters constrained
Δρ_max_, Δρ_min_ (e Å^−3^)	0.30, −0.32

Computer programs: *X-AREA* (Stoe & Cie, 2002 ▸), *X-RED* (Stoe & Cie, 2002 ▸), *SHELXT2017* (Sheldrick, 2015*a* ▸), *Mercury* (Macrae *et al.*, 2008 ▸), *SHELXL2018* (Sheldrick, 2015*b* ▸), *PLATON* (Spek, 2009 ▸) and *publCIF* (Westrip, 2010 ▸).

## supplementary crystallographic information

### Crystal data

**Table:**

C_18_H_16_N_2_O	*Z* = 2
*M**_r_* = 276.33	*F*(000) = 292
Triclinic, *P*1	*D*_x_ = 1.248 Mg m^−^^3^
*a* = 5.8479 (5) Å	Mo *K*α radiation, λ = 0.71073 Å
*b* = 8.5738 (7) Å	Cell parameters from 8216 reflections
*c* = 15.2439 (12) Å	θ = 2.5–30.7°
α = 80.693 (6)°	µ = 0.08 mm^−^^1^
β = 83.147 (7)°	*T* = 296 K
γ = 78.164 (7)°	Prism, colorless
*V* = 735.27 (11) Å^3^	0.27 × 0.20 × 0.06 mm

### Data collection

**Table:**

Stoe IPDS 2 diffractometer	2887 independent reflections
Radiation source: sealed X-ray tube, 12 x 0.4 mm long-fine focus	1471 reflections with *I* > 2σ(*I*)
Plane graphite monochromator	*R*_int_ = 0.086
Detector resolution: 6.67 pixels mm^-1^	θ_max_ = 26.0°, θ_min_ = 2.5°
rotation method scans	*h* = −7→7
Absorption correction: integration (X-RED32; Stoe & Cie, 2002)	*k* = −10→10
*T*_min_ = 0.966, *T*_max_ = 0.996	*l* = −18→18
9453 measured reflections	

### Refinement

**Table:**

Refinement on *F*^2^	Primary atom site location: dual space
Least-squares matrix: full	Secondary atom site location: difference Fourier map
*R*[*F*^2^ > 2σ(*F*^2^)] = 0.068	Hydrogen site location: inferred from neighbouring sites
*wR*(*F*^2^) = 0.208	H-atom parameters constrained
*S* = 1.05	*w* = 1/[σ^2^(*F*_o_^2^) + (0.0939*P*)^2^] where *P* = (*F*_o_^2^ + 2*F*_c_^2^)/3
2887 reflections	(Δ/σ)_max_ < 0.001
191 parameters	Δρ_max_ = 0.30 e Å^−^^3^
84 restraints	Δρ_min_ = −0.32 e Å^−^^3^

### Special details

**Table:**

Geometry. All esds (except the esd in the dihedral angle between two l.s. planes) are estimated using the full covariance matrix. The cell esds are taken into account individually in the estimation of esds in distances, angles and torsion angles; correlations between esds in cell parameters are only used when they are defined by crystal symmetry. An approximate (isotropic) treatment of cell esds is used for estimating esds involving l.s. planes.

### Fractional atomic coordinates and isotropic or equivalent isotropic displacement parameters (Å^2^)

**Table:**

	*x*	*y*	*z*	*U*_iso_*/*U*_eq_	
O1	0.9692 (4)	0.6487 (3)	0.90252 (15)	0.0776 (7)	
N1	0.7762 (4)	0.4423 (3)	0.94429 (17)	0.0653 (7)	
H1	0.848007	0.419860	0.992137	0.078*	
N2	0.6315 (4)	0.3435 (3)	0.93530 (17)	0.0621 (7)	
C10	0.8227 (5)	0.5725 (4)	0.8874 (2)	0.0627 (8)	
C13	0.3636 (6)	0.2661 (4)	0.8525 (2)	0.0728 (8)	
C11	0.5151 (5)	0.3799 (4)	0.8641 (2)	0.0594 (8)	
C5	0.6414 (6)	0.7618 (4)	0.6536 (2)	0.0668 (9)	
C9	0.6964 (5)	0.6101 (4)	0.8080 (2)	0.0630 (8)	
C12	0.5470 (5)	0.5134 (4)	0.7992 (2)	0.0671 (9)	
H12	0.463050	0.535285	0.749060	0.080*	
C2	0.4368 (7)	0.7711 (4)	0.4949 (2)	0.0738 (10)	
C18	0.2108 (6)	0.2984 (4)	0.7881 (2)	0.0750 (8)	
H18	0.200948	0.394224	0.748873	0.090*	
C3	0.3212 (7)	0.8506 (5)	0.5622 (3)	0.0806 (11)	
H3	0.170292	0.909110	0.555296	0.097*	
C17	0.0695 (6)	0.1901 (5)	0.7802 (3)	0.0812 (9)	
H17	−0.034226	0.214814	0.736029	0.097*	
C8	0.7452 (7)	0.7504 (4)	0.7414 (2)	0.0793 (11)	
H8A	0.913650	0.742443	0.729872	0.095*	
H8B	0.682622	0.848692	0.766918	0.095*	
C16	0.0804 (7)	0.0510 (5)	0.8351 (3)	0.0895 (10)	
H16	−0.016404	−0.019967	0.829942	0.107*	
C4	0.4200 (7)	0.8474 (4)	0.6402 (2)	0.0770 (10)	
H4	0.335386	0.904240	0.684478	0.092*	
C6	0.7593 (6)	0.6817 (5)	0.5855 (3)	0.0819 (11)	
H6	0.910392	0.623176	0.591920	0.098*	
C7	0.6572 (7)	0.6867 (5)	0.5077 (3)	0.0870 (11)	
H7	0.741083	0.631025	0.462885	0.104*	
C1	0.3250 (8)	0.7769 (6)	0.4096 (3)	0.1041 (14)	
H1A	0.164486	0.831458	0.415749	0.156*	
H1B	0.330986	0.669253	0.398093	0.156*	
H1C	0.408494	0.833710	0.360869	0.156*	
C14	0.3757 (8)	0.1221 (5)	0.9057 (3)	0.1055 (11)	
H14	0.482967	0.094249	0.948657	0.127*	
C15	0.2333 (8)	0.0152 (6)	0.8979 (3)	0.1111 (11)	
H15	0.243396	−0.081609	0.936290	0.133*	

### Atomic displacement parameters (Å^2^)

**Table:**

	*U*^11^	*U*^22^	*U*^33^	*U*^12^	*U*^13^	*U*^23^
O1	0.0901 (16)	0.0843 (16)	0.0741 (15)	−0.0425 (13)	−0.0351 (12)	−0.0038 (12)
N1	0.0704 (16)	0.0782 (18)	0.0577 (16)	−0.0304 (14)	−0.0247 (13)	−0.0051 (14)
N2	0.0635 (15)	0.0736 (18)	0.0579 (16)	−0.0271 (13)	−0.0179 (12)	−0.0074 (13)
C10	0.0695 (19)	0.069 (2)	0.059 (2)	−0.0260 (16)	−0.0192 (15)	−0.0100 (16)
C13	0.0799 (18)	0.0761 (18)	0.0732 (19)	−0.0356 (16)	−0.0284 (15)	−0.0002 (15)
C11	0.0597 (18)	0.066 (2)	0.0576 (19)	−0.0200 (15)	−0.0160 (15)	−0.0061 (16)
C5	0.078 (2)	0.0570 (19)	0.073 (2)	−0.0278 (17)	−0.0288 (18)	0.0040 (17)
C9	0.0674 (19)	0.066 (2)	0.062 (2)	−0.0214 (16)	−0.0219 (15)	−0.0037 (16)
C12	0.0689 (19)	0.074 (2)	0.067 (2)	−0.0271 (17)	−0.0306 (16)	−0.0010 (17)
C2	0.092 (3)	0.077 (2)	0.061 (2)	−0.036 (2)	−0.0167 (19)	−0.0020 (18)
C18	0.0798 (18)	0.0817 (18)	0.0737 (18)	−0.0301 (15)	−0.0258 (15)	−0.0083 (15)
C3	0.078 (2)	0.086 (3)	0.078 (3)	−0.008 (2)	−0.028 (2)	−0.007 (2)
C17	0.0827 (18)	0.093 (2)	0.083 (2)	−0.0327 (16)	−0.0299 (16)	−0.0198 (16)
C8	0.096 (3)	0.079 (2)	0.075 (2)	−0.043 (2)	−0.034 (2)	0.0059 (19)
C16	0.098 (2)	0.093 (2)	0.095 (2)	−0.0501 (18)	−0.0263 (17)	−0.0138 (17)
C4	0.087 (3)	0.080 (2)	0.068 (2)	−0.012 (2)	−0.0180 (19)	−0.0154 (19)
C6	0.068 (2)	0.083 (3)	0.095 (3)	−0.0052 (19)	−0.020 (2)	−0.015 (2)
C7	0.090 (3)	0.102 (3)	0.073 (3)	−0.017 (2)	−0.006 (2)	−0.026 (2)
C1	0.132 (4)	0.117 (3)	0.077 (3)	−0.044 (3)	−0.038 (2)	−0.006 (2)
C14	0.121 (2)	0.101 (2)	0.110 (2)	−0.0590 (19)	−0.0567 (19)	0.0217 (18)
C15	0.128 (2)	0.102 (2)	0.119 (2)	−0.0644 (19)	−0.0481 (19)	0.0192 (19)

### Geometric parameters (Å, º)

**Table:**

O1—C10	1.241 (3)	C18—H18	0.9300
N1—N2	1.347 (3)	C3—C4	1.378 (5)
N1—C10	1.352 (4)	C3—H3	0.9300
N1—H1	0.8600	C17—C16	1.336 (5)
N2—C11	1.311 (4)	C17—H17	0.9300
C10—C9	1.449 (4)	C8—H8A	0.9700
C13—C14	1.357 (5)	C8—H8B	0.9700
C13—C18	1.364 (4)	C16—C15	1.344 (6)
C13—C11	1.488 (4)	C16—H16	0.9300
C11—C12	1.415 (4)	C4—H4	0.9300
C5—C4	1.372 (5)	C6—C7	1.381 (5)
C5—C6	1.375 (5)	C6—H6	0.9300
C5—C8	1.515 (4)	C7—H7	0.9300
C9—C12	1.353 (4)	C1—H1A	0.9600
C9—C8	1.496 (4)	C1—H1B	0.9600
C12—H12	0.9300	C1—H1C	0.9600
C2—C3	1.359 (5)	C14—C15	1.386 (5)
C2—C7	1.361 (5)	C14—H14	0.9300
C2—C1	1.512 (5)	C15—H15	0.9300
C18—C17	1.391 (4)		
			
N2—N1—C10	128.0 (2)	C16—C17—H17	119.5
N2—N1—H1	116.0	C18—C17—H17	119.5
C10—N1—H1	116.0	C9—C8—C5	113.7 (2)
C11—N2—N1	116.5 (3)	C9—C8—H8A	108.8
O1—C10—N1	120.9 (3)	C5—C8—H8A	108.8
O1—C10—C9	123.9 (3)	C9—C8—H8B	108.8
N1—C10—C9	115.1 (2)	C5—C8—H8B	108.8
C14—C13—C18	116.8 (3)	H8A—C8—H8B	107.7
C14—C13—C11	120.7 (3)	C17—C16—C15	119.1 (3)
C18—C13—C11	122.5 (3)	C17—C16—H16	120.5
N2—C11—C12	121.3 (3)	C15—C16—H16	120.5
N2—C11—C13	116.2 (3)	C5—C4—C3	121.0 (4)
C12—C11—C13	122.4 (3)	C5—C4—H4	119.5
C4—C5—C6	117.0 (3)	C3—C4—H4	119.5
C4—C5—C8	121.1 (4)	C5—C6—C7	121.1 (4)
C6—C5—C8	121.9 (3)	C5—C6—H6	119.4
C12—C9—C10	117.4 (3)	C7—C6—H6	119.4
C12—C9—C8	125.0 (3)	C2—C7—C6	121.7 (4)
C10—C9—C8	117.5 (2)	C2—C7—H7	119.1
C9—C12—C11	121.7 (3)	C6—C7—H7	119.1
C9—C12—H12	119.1	C2—C1—H1A	109.5
C11—C12—H12	119.1	C2—C1—H1B	109.5
C3—C2—C7	117.0 (3)	H1A—C1—H1B	109.5
C3—C2—C1	121.2 (4)	C2—C1—H1C	109.5
C7—C2—C1	121.8 (4)	H1A—C1—H1C	109.5
C13—C18—C17	120.9 (4)	H1B—C1—H1C	109.5
C13—C18—H18	119.6	C13—C14—C15	122.0 (4)
C17—C18—H18	119.6	C13—C14—H14	119.0
C2—C3—C4	122.2 (4)	C15—C14—H14	119.0
C2—C3—H3	118.9	C16—C15—C14	120.1 (4)
C4—C3—H3	118.9	C16—C15—H15	119.9
C16—C17—C18	121.0 (3)	C14—C15—H15	119.9
			
C10—N1—N2—C11	−2.3 (5)	C1—C2—C3—C4	−179.7 (4)
N2—N1—C10—O1	−176.7 (3)	C13—C18—C17—C16	−0.4 (6)
N2—N1—C10—C9	1.2 (5)	C12—C9—C8—C5	−9.4 (6)
N1—N2—C11—C12	1.9 (5)	C10—C9—C8—C5	169.2 (3)
N1—N2—C11—C13	177.4 (3)	C4—C5—C8—C9	90.3 (4)
C14—C13—C11—N2	−9.6 (5)	C6—C5—C8—C9	−86.8 (4)
C18—C13—C11—N2	171.6 (3)	C18—C17—C16—C15	−1.0 (7)
C14—C13—C11—C12	165.7 (4)	C6—C5—C4—C3	0.7 (5)
C18—C13—C11—C12	−13.0 (5)	C8—C5—C4—C3	−176.5 (3)
O1—C10—C9—C12	178.0 (3)	C2—C3—C4—C5	−0.5 (6)
N1—C10—C9—C12	0.2 (5)	C4—C5—C6—C7	−0.6 (5)
O1—C10—C9—C8	−0.7 (5)	C8—C5—C6—C7	176.6 (3)
N1—C10—C9—C8	−178.5 (3)	C3—C2—C7—C6	0.0 (6)
C10—C9—C12—C11	−0.4 (5)	C1—C2—C7—C6	179.9 (4)
C8—C9—C12—C11	178.2 (3)	C5—C6—C7—C2	0.2 (6)
N2—C11—C12—C9	−0.7 (5)	C18—C13—C14—C15	−2.7 (7)
C13—C11—C12—C9	−175.9 (3)	C11—C13—C14—C15	178.5 (4)
C14—C13—C18—C17	2.2 (6)	C17—C16—C15—C14	0.5 (7)
C11—C13—C18—C17	−179.0 (3)	C13—C14—C15—C16	1.4 (8)
C7—C2—C3—C4	0.1 (6)		

### Hydrogen-bond geometry (Å, º)

**Table:**

*D*—H···*A*	*D*—H	H···*A*	*D*···*A*	*D*—H···*A*
N1—H1···O1^i^	0.86	1.98	2.836 (3)	175
C14—H14···N2	0.93	2.43	2.764 (3)	101

Symmetry code: (i) −*x*+2, −*y*+1, −*z*+2.
